# Supplementary material for: Barriers and Facilitators to Conversation: A Qualitative Exploration of the Experiences of People with Parkinson’s and Their Close Communication Partners
Source: Brain Sci. 2022 Jul 19;12(7):944. doi: 10.3390/brainsci12070944 (PMC9321478; doi:10.3390/brainsci12070944)
Supplement: Supplementary file 1 [file brainsci-12-00944-s001.zip › brainsci-1808917-supplementary.pdf]

Semi-Structured Topic Guide Questions and Probes

Joint Interview Guide

Lead Question 1

Can you describe things that make your conversations more difficult?

Follow-Up Questions

- What do you see as the most challenging part of having conversations?
- Can you think of a time when you had a challenging experience when having a conversation and try to describe how it felt?

Clarification and probe example questions (Following the participants lead to further explore their responses)

- So <Name>, you spoke about XXX, have I understood that correctly?
- <Name> you mentioned XXX earlier, can you tell me a bit more about that?
- Can you help me understand X?

Lead Question 2

Can you describe things that you do to improve or repair the conversations you have?

Follow-Up Questions

- What do you see as your most successful facilitator when having a conversation?
- Can you think of a time when you had a successful conversation experience and try to describe it?
- Are there particular things you do to repair or improve your conversations?
- What do you do when X (barrier) happens?
- Can you give any examples of how you or your partner have overcome challenges when having conversations?
- If you could give advice to someone about what to do or not to do when

having a conversation with you to help, what would that be?

Clarification and probe example questions (Following the participants lead to further explore their responses)

- So <Name>, you spoke about XXX, have I understood that correctly?
- <Name> you mentioned XXX earlier, can you tell me a bit more about that?
- Can you help me understand X?

Individual Interview Guide

Lead Question

I wanted to speak with you individually to see if there is anything further you would like to add or clarify about what you see as difficult or helpful when having a conversation with your partner. I will ask you to describe barriers and facilitators you experience due to your conversation difficulties in difficult social situations.

Follow-Up Questions

- Do you have any comments following the joint interview?
- I noticed during the joint interview that you seemed to have more to say about X. Would you like to talk about that a little more?
- Your partner said X – would you like to add anything further?
